# Supplementary material for: Core outcome sets for spinal and associated limb, trunk, abdomen or pelvic pain: A systematic review
Source: PLoS One. 2025 Dec 12;20(12):e0338184. doi: 10.1371/journal.pone.0338184 (PMC12700389; doi:10.1371/journal.pone.0338184)
Supplement: S1 File — (DOCX) [file pone.0338184.s001.docx]

Supplementary File 1: Quality assessment

| Reporting Items | Chen et al, 2019 | Chiarotto et al, 2018 | Cieza et al, 2004 | Clement et al, 2015 | Deyo et a, 1998 | Deyo et al, 2014 | de Kleuver et al, 2017 | Pincus et al, 2008 | Rebbeck et al, 2007 | Sterling et al, 2023 | Verburg et al, 2019 | Verburg et al, 2021 | White et al, 2004 |
| --- | --- | --- | --- | --- | --- | --- | --- | --- | --- | --- | --- | --- | --- |
| Rationale | | | | | | | | | | | | | |
| *Justification.* The choice of the Delphi technique as a method of systematically  collating expert consultation and building consensus needs to be well justified. | 1 | 1 | 0 | 1 | 0 | 0.5 | 0.5 | 0.5 | 0 | 1 | 1 | 0.5 | 0 |
| Planning and Design | | | | | | | | | | | | | |
| *Planning and process.* The Delphi technique is a flexible method and can be adjusted  to the respective research aims and purposes. Any modifications should be justified by  a rationale and be applied systematically and rigorously. | 1 | 1 | 0 | 1 | 0 | 0 | 1 | 0.5 | 0 | 1 | 1 | 0 | 0 |
| *Definition of consensus.* Unless not reasonable due to the explorative nature of the  study, an a priori criterion for consensus should be defined. This includes a clear and  transparent guide for action on (a) how to proceed with certain items or topics in  the next survey round, (b) the required threshold to terminate the Delphi process and  (c) procedures to be followed when consensus is (not) reached after one or more  Iterations. | 1 | 1 | 0 | 0.5 | 0 | 0 | 1 | 1 | 0 | 1 | 1 | 0 | 0 |
| Study Conduct | | | | | | | | | | | | | |
| *Informational input.* All material provided to the expert panel at the outset of the  project and throughout the Delphi process should be carefully reviewed and piloted in  advance in order to examine the effect on experts’ judgements and to prevent bias. | 0 | 1 | 0 | 0 | 0 | 0 | 0 | 0 | 0 | 1 | 0 | 0 | 0 |
| *Prevention of bias.* Researchers need to take measures to avoid directly or indirectly  influencing the experts’ judgements. If one or more members of the research team  have a conflict of interest, entrusting an independent researcher with the main  coordination of the Delphi study is advisable. | 1 | 1 | 0 | 0 | 0 | 0 | 0 | 0 | 0 | 1 | 0 | 0 | 0 |
| *Interpretation and processing of results.* Consensus does not necessarily imply the  ‘correct’ answer or judgement; (non)consensus and stable disagreement provide  informative insights and highlight differences in perspectives concerning the topic in  question. | 1 | 1 | 1 | 1 | 1 | 1 | 1 | 1 | 0 | 1 | 1 | 1 | 0 |
| *External validation.* It is recommended to have the final draft of the resulting  guidance reviewed and approved by an external board or authority before publication  and dissemination. | 1 | 1 | 0.5 | 0 | 0 | 0 | 0 | 0 | 0 | 1 | 1 | 0 | 0 |
| Reporting | | | | | | | | | | | | | |
| *Purpose and rationale*. The purpose of the study should be clearly defined and  demonstrate the appropriateness of the use of the Delphi technique as a method to  achieve the research aim. A rationale for the choice of the Delphi technique as the  most suitable method needs to be provided. | 1 | 1 | 0.5 | 1 | 0 | 0.5 | 1 | 1 | 0 | 1 | 1 | 0 | 0 |
| *Expert panel.* Criteria for the selection of experts and transparent information on recruitment of the expert panel, sociodemographic details including information on expertise regarding the topic in question, (non)response and response rates over the ongoing iterations should be reported. | 1 | 0.5 | 0 | 0 | 0 | 1 | 1 | 0 | 0 | 1 | 1 | 0 | 0 |
| *Description of the methods.* The methods employed need to be comprehensible; this includes information on preparatory steps (How was available evidence on the topic in question synthesised?), piloting of material and survey instruments, design of the survey instrument(s), the number and design of survey rounds, methods of data analysis, processing and synthesis of experts’ responses to inform the subsequent survey round and methodological decisions taken by the research team throughout the process. | 1 | 1 | 0 | 0 | 0 | 0 | 1 | 0 | 0 | 1 | 1 | 0 | 0 |
| *Procedure.* Flow chart to illustrate the stages of the Delphi process, including a preparatory phase, the actual ‘Delphi rounds’, interim steps of data processing and analysis, and concluding steps. | 1 | 1 | 0 | 0 | 0 | 0 | 1 | 0 | 0 | 0 | 0 | 0 | 0 |
| *Definition and attainment of consensus.* It needs to be comprehensible to the reader how consensus was achieved throughout the process, including strategies to deal with non-consensus. | 1 | 1 | 0 | 0 | 0 | 0 | 1 | 0 | 0 | 1 | 1 | 0 | 0 |
| *Results*. Reporting of results for each round separately is highly advisable in order to make the evolving of consensus over the rounds transparent. This includes figures showing the average group response, changes between rounds, as well as any modifications of the survey instrument such as deletion, addition or modification of survey items based on previous rounds. | 1 | 1 | 0 | 0 | 0 | 0 | 1 | 0 | 0 | 0 | 1 | 0 | 0 |
| *Discussion of limitations.* Reporting should include a critical reflection of potential limitations and their impact of the resulting guidance. | 1 | 1 | 0.5 | 1 | 0 | 0 | 1 | 1 | 0 | 1 | 1 | 1 | 0 |
| *Adequacy of conclusions.* The conclusions should adequately reflect the outcomes of the Delphi study with a view to the scope and applicability of the resulting practice guidance. | 1 | 0.5 | 0 | 1 | 0 | 1 | 1 | 0 | 0 | 1 | 1 | 0 | 0 |
| *Publication and dissemination*. The resulting guidance should be clearly identifiable  from the publication, including recommendations for transfer into practice and  implementation. A dissemination plan should include endorsement of the  guidance by professional associations and health care authorities to facilitate  implementation. | 1 | 1 | 0 | 1 | 1 | 1 | 1 | 0 | 0.5 | 1 | 1 | 0 | 0.5 |
| **Total /16** | 15 | 15 | 2.5 | 7.5 | 2 | 5 | 12.5 | 5 | 0.5 | 14 | 13 | 2.5 | 0.5 |
