# Supplementary material for: Core outcome sets for spinal and associated limb, trunk, abdomen or pelvic pain: A systematic review
Source: PLoS One. 2025 Dec 12;20(12):e0338184. doi: 10.1371/journal.pone.0338184 (PMC12700389; doi:10.1371/journal.pone.0338184)
Supplement: Attachment — Submitted filename: Response to reviewers.docx [file pone.0338184.s004.docx]

11^th^ November 2025

Priti Chaudhary, M.S.
Academic Editor
PLOS ONE

PONE-D-25-44505

Core Outcome Sets for Spinal and Associated Limb, Trunk, Abdomen or Pelvic Pain: a systematic review

Dear Priti Chaudhary,

Thank you for inviting us to submit a revised version of our manuscript addressing the points raised within the review process. Please find attached alongside this letter a copy of the revised manuscript with track changes and the clean manuscript as requested.

Please see tabulated below an outline of each change made as raised in the reviewer comments, or our reasoning as to why it has not been possible to address a comment.

All authors have approved the edited manuscript and submission to PLOS One. We appreciate your time and thank you for considering this manuscript for publication. Should you have any further questions please do not hesitate to contact us.

Kind Regards,

Tim Noblet PhD

On behalf of all authors

| Editors/Reviewers Comments | Changes made or Reason(s) for not making changes |
| --- | --- |
| Journal Requirements | |
| We note that your Data Availability Statement is currently as follows: All relevant data are within the manuscript and its Supporting Information files.    Please confirm at this time whether or not your submission contains all raw data required to replicate the results of your study. Authors must share the “minimal data set” for their submission. | We confirm that our submission contains all raw data required to replicate the results of the study. The complete minimal data set is provided within the manuscript and its Supporting Information files. |
| We notice that your supplementary files are included in the manuscript file. Please remove them and upload them with the file type 'Supporting Information'. Please ensure that each Supporting Information file has a legend listed in the manuscript after the references list. | Thank you for your comment. This has been completed. |
| If the reviewer comments include a recommendation to cite specific previously published works, please review and evaluate these publications to determine whether they are relevant and should be cited. There is no requirement to cite these works unless the editor has indicated otherwise. | N/A |
| Please review your reference list to ensure that it is complete and correct. If you have cited papers that have been retracted, please include the rationale for doing so in the manuscript text, or remove these references and replace them with relevant current references. Any changes to the reference list should be mentioned in the rebuttal letter that accompanies your revised manuscript. If you need to cite a retracted article, indicate the article’s retracted status in the References list and also include a citation and full reference for the retraction notice. | Thank you for your comment. The reference list has been thoroughly reviewed and verified. It is complete, accurate, and does not include any retracted articles. No additional references have been added or removed. |
| Reviewer 1 | |
| Abstract Percentages are reported but the denominator is not shown. Always report n (%) so readers can interpret percentages correctly.  While recommending a Delphi study is sensible, the abstract should present this as a logical next step rather than a definitive solution. | Thank you for your comment. Percentages have been revised to include the corresponding denominators, now reported as n (%) as recommended.  The abstract now reads:  *“A Delphi study to develop an internationally agreed ‘Essential Universal Set’ for spinal pain, inclusive of multidimensional biopsychosocial domains, is a sound next step.”*  To show that a Delphi is a logical next step rather than a definitive solution. |
| Introduction The first two paragraphs (lines ~66–89) both describe global burden, heterogeneity, and costs. These could be combined and streamlined for concision.  The figure “790 million people” should be tied to specific Global Burden of Disease years/metrics. Readers expect exact sources and dates.  The Introduction notes lack of consensus on a single COS but does not sufficiently review prior COS efforts. A brief overview of past efforts would help clarify novelty.  Some method-like statements in the Introduction such as breadth of research approaches and study types could be trimmed or relocated to Methods. The Introduction should stay focused on background, gap, and aim. | We appreciate the suggestion to streamline the opening paragraphs. However, both paragraphs have been intentionally retained as they serve distinct yet complementary purposes in framing the study.  The first paragraph establishes the global significance, burden, and societal impact of spinal pain, providing essential context for why consensus on outcome measurement is needed. It foregrounds the scale, disability burden, and economic consequences that justify the study’s relevance and urgency.  The second paragraph transitions to highlight the heterogeneity and multidisciplinary nature of spinal pain research, demonstrating the breadth of contributing disciplines, methodologies, and populations studied. This diversity directly underpins the rationale for developing a unified Core Outcome Set (COS) to enable comparability and synthesis across this fragmented evidence base.  Together, these paragraphs lay a coherent foundation linking the magnitude of the problem to the need for methodological standardisation, aligning with the study’s aim and objectives.  Thank you for your helpful comments.  All figures and statistics, including the “790 million people” estimate, are now clearly referenced in accordance with *PLOS ONE* guidelines, including the relevant Global Burden of Disease source and year. The systematic review comprehensively examines all available Core Outcome Sets (COSs) within the Results section, consistent with the stated aim and objectives of the study.  For clarity we have added the following into the introduction:  *“Several core outcome sets (COSs) have been developed for spinal pain and related musculoskeletal conditions over the past two decades. Notable examples include outcome sets for low back pain (21, 22) whiplash-associated disorders (23) and spinal deformity surgery (24). However, these efforts have generally focused on isolated anatomical regions or specific conditions, leading to fragmentation in measurement approaches. To date, no systematic review has comprehensively mapped and appraised COSs across the full spectrum of spinal pain and its common referred pain presentations.”*  We would like to clarify that the discussion of diverse research approaches in spinal pain is not intended to justify the methodology of this review. Rather, it serves to contextualise the fragmented landscape of outcome measurement in spinal pain research. The variety of approaches has contributed to the development of multiple COSs, often tailored to specific settings or aims. Including this background supports the rationale for our review: to synthesise and update existing COSs for use in contemporary research and clinical practice. |
| Methods The Methods do not state whether non-English records were eligible. | This information can be found in Fig 2: Eligibility Criteria. All languages were included. |
| Discussion Much of the Discussion re-iterates results rather than synthesizing them into deeper interpretation. The authors should move beyond “what” was found to explain why patterns exist and what that means for implementation.  Beyond identifying domains, a staged process is needed: review psychometric evidence for candidate instruments, test feasibility across settings, and pilot implementation before finalizing a minimal core set. | Thank you for this constructive feedback. We acknowledge the suggestion to extend the Discussion with deeper interpretation and concrete short-term recommendations for researchers.  The following sections have been added to address the reviewer’s comments:  *“The consistent prioritisation of domains such as pain intensity and physical function across COSs likely reflects the long-standing biomedical orientation of spinal pain research and the preference for outcomes that are easily measured, clinically interpretable, and responsive to intervention. Conversely, the limited inclusion of psychological, social, and biological domains may result from practical constraints, disciplinary silos in COS development, and concerns regarding feasibility in clinical settings. These patterns suggest that while core domains remain stable, broader biopsychosocial factors, essential to precision and person-centred care, are underrepresented, limiting the comprehensiveness of existing COSs.” Page 50-51*  *“To enhance adoption and relevance across settings, COS implementation must be accompanied by a staged, evidence-informed process. First, robust psychometric appraisal of candidate instruments is needed to ensure reliability and validity across spinal populations. Second, feasibility testing in routine clinical environments (across primary, secondary, and tertiary care) is required to determine acceptability and integration potential. Finally, pilot implementation studies should be conducted to evaluate the COS’s impact on clinical decision-making, patient experience, and research data quality. Without this staged approach, there is a risk that even well-constructed COSs may fail to achieve meaningful uptake or influence practice.” Page 52-53*  The Discussion currently reflects the available evidence base and provides logical, methodologically sound next steps consistent with contemporary approaches in this field. |
| Reviewer 2 | |
| The manuscript was interesting and good written and discussed. Introduction was good written. Materials and methods were good designed. Results were good described. Discussion was good written and include the conclusion of the study. | Thank you for your review. We welcome your comments and feedback. |
